# Security importance of edge-IoT ecosystem: An ECC-based authentication scheme

**DOI:** 10.1371/journal.pone.0322131

**Published:** 2025-06-04

**Authors:** Naif Alzahrani

**Affiliations:** College of Computer Science and Engineering, University of Jeddah, Jeddah, Saudi Arabia; Maulana Abul Kalam Azad University of Technology West Bengal, INDIA

## Abstract

Despite the many outstanding benefits of cloud computing, such as flexibility, accessibility, efficiency, and cost savings, it still suffers from potential data loss, security concerns, limited control, and availability issues. The experts introduced the edge computing paradigm to perform better than cloud computing for the mentioned issues and challenges because it is directly connected to the Internet-of-Things (IoT), sensors, and wearables in a decentralized manner to distribute processing power closer to the data source, rather than relying on a central cloud server to handle all computations; this allows for faster data processing and reduced latency by processing data locally at the ‘edge’ of the network where it’s generated. However, due to the resource-constrained nature of IoT, sensors, or wearable devices, the edge computing paradigm endured numerous data breaches due to sensitive data proximity, physical tampering vulnerabilities, and privacy concerns related to user-near data collection, and challenges in managing security across a large number of edge devices. Existing authentication schemes didn’t fulfill the security needs of the edge computing paradigm; they either have design flaws, are susceptible to various known threats—such as impersonation, insider attacks, denial of service (DoS), and replay attacks—or experience inadequate performance due to reliance on resource-intensive cryptographic algorithms, like modular exponentiations. Given the pressing need for robust security mechanisms in such a dynamic and vulnerable edge-IoT ecosystem, this article proposes an ECC-based robust authentication scheme for such a resource-constrained IoT to address all known vulnerabilities and counter each identified threat. The proof of correctness of the proposed protocol has been scrutinized through a well-known and widely used Real-Or-Random (RoR) model, ProVerif validation, and attacks’ discussion, demonstrating the thoroughness of the proposed protocol. The performance metrics have been measured by considering computational time complexity, communication cost, and storage overheads, further reinforcing the confidence in the proposed solution. The comparative analysis results demonstrated that the proposed ECC-based authentication protocol is 90.05% better in terms of computation cost, 62.41% communication cost, and consumes 67.42% less energy compared to state-of-the-art schemes. Therefore, the proposed protocol can be recommended for practical implementation in the real-world edge-IoT ecosystem.

## Introduction

Cloud computing providers handle software updates, patches, and system maintenance, ensuring that applications and infrastructure are always up-to-date without requiring user manual intervention. They also offer backup and disaster recovery solutions, resources management, applications, and data from a single location. Despite the many outstanding benefits of cloud computing, it has many drawbacks, including excessive latency, inadequate bandwidth, excessive energy consumption, dangers to data security and privacy, etc, and is not suitable directly for IoT applications. However, the edge-IoT ecosystem, focusing on decentralized computing at the network’s edge, is poised to revolutionize data processing and management, offering a promising future for IoT applications [1].

The self-change (decentralization) of the edge computing paradigm is a necessary evolution for several applications, including industrial automation and healthcare. It promises to improve responsiveness, lower latency, maximize resource usage, and increase efficiency and performance [2]. This ecosystem processes data close to its source to minimize latency, bandwidth usage, and real-time analytics, which is only possible by integrating IoT devices and the edge server, lessening the need for centralized cloud resources. One key aspect of this integration is ‘job offloading balance,’‘ a technique that distributes computational tasks between the IoT devices and the edge server, thereby significantly improving system performance [3]. In addition to limiting the resources on the user/IoT side, the edge server allows on-device data processing, which speeds up decision-making and enhances security [4]. Innovative resource management techniques are necessary because an edge server frequently lacks sophisticated analytics processing capacity [5]. Despite the many benefits of the edge-IoT ecosystem and its decentralized nature, it presents interoperability, security, and privacy issues that can only be mitigated by designing a robust, secure, and lightweight authentication system to audit the protection of critical information.

As stated, the edge computing paradigm has drawn interest from various sources by bringing processing and storage resources closer to IoT applications, significantly reducing processing time [6]. This is partially achieved by the role of edge nodes, which can perform some operations directly, reducing the pressure on cloud computing, which in turn can benefit by protecting IoT/user data security and ensuring the controllability of sensitive data [7]. It also improves the IoT/user response time and reduces network latency [8]. With these capabilities, the edge-IoT ecosystem has the potential to outperform better than other computer paradigms, leading to an exciting future for IoT technology. Therefore, the security of the edge-IoT ecosystem is of utmost importance. Making it more advanced and safe against numerous threats is crucial, as the attacker can fabricate the user/sensor/IoT side data and influence the decision and control outcomes [9]. Attackers might hack edge nodes to conduct malicious actions like a denial of service (DoS) and insider attacks to stop the authorized user/IoT from using the services of the edge server. So, it is challenging to address these concerns and maintain edge-IoT ecosystem security because they hold and process data in many application areas, including smart homes, weather forecasting, and e-healthcare systems [10]. These application areas of the edge-IoT ecosystem are more sensitive and transmit voluminous data via a hostile environment that exposes them to the possibility of being compromised by attackers. Given the sharp increase in attacks directed at edge computing infrastructures, it is critical to create robust security measures against these risks in recent years [11].

Furthermore, IoT’s resource-constrained and limited performance characteristics introduce additional challenges in developing and implementing security measures. The extensive number of sensors, wearables, and servers in the architecture can significantly affect IoT applications, especially when security techniques impose a heavy computational burden [12]. Therefore, continuous efforts are essential to tackle these challenges and urgently create a secure ecosystem, highlighting the importance of balancing performance and security trade-offs. With this critical task in mind, the key contributions of this research are as follows:

- To introduce a security mechanism for the edge-IoT ecosystem by utilizing a lightweight cryptographic method called ECC in which the sensor/IoT devices dynamically prove their authenticity in the decentralized environment and generally show resistance to all known threats, and specifically impersonation, insider, denial of service (DoS), and replay attacks.
- A Real-Or-Random (RoR) model, ProVerif validation, and informal discussions are robustly scrutinizing the security of the proposed authentication scheme. This priority of security analysis through these well-known methods provides reassurance of the proposed protocol’s robustness.
- To measure the performance by considering storage, communication, and computation costs.
- To check its efficacy and robustness, the proposed protocol will be compared with state-of-the-art protocols in terms of computational time complexity and communication cost.
- To ensure a balance among the performance-security trade-offs is challenging, as these are contradictory features, and a change in one inversely affects the other, which is often missing in existing works available in the literature.

The remainder of the article is structured as follows: the preliminaries and background section demonstrates the foundation concepts and methods utilized in this research article, the related works section overviews the existing literature and identifies loophole(s) in it, and the system model section presents the proposed network model, threats to the system in the threat model, and fixed design goals for demonstrating the proposed authentication protocol. In the proposed authentications scheme section of the article, the ECC-based protocol for the edge-IoT ecosystem has been presented utilizing the SHA256 algorithm; in the analysis and discussion section, the proof of correctness of the proposed authentication scheme has been conducted both formally and informally, while in the performance analysis section, the efficiency and efficacy of the proposed protocol have been measured by considering computation, communication, and storage costs and then compared the proposed protocol with prior works, while in the conclusion section described what we have concluded while conducting this research work.

## Preliminaries and background

This section of the article presents the foundation knowledge pertaining to conducting this research work compressively. These preliminary concepts include IoT, ECC, and associated terminologies and definitions which are explained one by one as follows:

### Edge-IoT ecosystem

Edge-IoT Ecosystem The edge-IoT ecosystem [13] is a complex web of interconnected layers, including the device, node, and cloud layers. These layers work in tandem to bring the virtual and real worlds together, each with its unique role and challenges.

#### Device layer.

This layer includes all devices, such as sensors, wearable technology, smart meters, cell phones, and video security cameras. These devices often process and transfer data from physical objects/devices to an edge server. These IoT devices have limited battery, processing, storage, and communication capabilities. The IoT device layer connects the real and virtual worlds to an edge server [13].

#### Node-layer.

A crucial part of the IoT, the edge node layer connects most IoT devices, such as smart thermostats, sensors, wearables, smartphones, smart watches, and other network-enable accessories. Heterogeneous networks connect all these wearables into the edge nodes to facilitate smooth transitions to the ecosystem. The voluminous data produced by these wearables can be processed and transmit control flows back to the edge node. The job offloading to the edge node is in full control until it reaches cloud servers if the task complexity exceeds the computation capabilities of the edge node layer [13].

#### Cloud layer.

The cloud server layer, which houses the centralized computing unit, plays a pivotal role in the IoT architecture. It is here that tasks with high complexity are offloaded from the edge nodes, thanks to the cloud servers’ strong processing capacity and ample data storage. This layer handles the highest degree of authentication, processing, and integration, making it a crucial part of the ecosystem [13].

The current research does not delve into the cloud layer, as the primary issue is concentrated in the device/node layers. However, these resource-constrained layers with limited processing capabilities and low bandwidth are vulnerable to exploitation by adversaries. The potential for an adversary to seize control of the connection between the device layer and edge layer, thereby disrupting the broadcast of sensitive information, underscores the urgency of our research.

### IoT architecture

The Internet of Things (IoT) is a robust tool in today’s technological landscape, comprising three main layers: application, middleware, and network layers. Each layer can be described as follows:

#### Application layer.

As the topmost layer, the application layer plays a vital role in the practical implementation of IoT by processing data for various uses. Data visualization in this layer utilizes graphs, flowcharts, business models, and other formats, with applications ranging from smart homes and intelligent vehicles to smart cities and automated car parking systems. However, this layer is susceptible to various attacks, including phishing, buffer overflow, denial-of-service (DoS), cross-site scripting attacks, and concerns regarding data privacy [14].

#### Middleware layer.

The middleware layer serves as more than just a line between the network and application layers in the IoT architecture; it acts as a critical link that manages the implementation of vendor-specific services tailored to handle various types of IoT node data. This layer simplifies the management, pre-processing, and archiving of the data generated by IoT nodes. Nonetheless, it is a prime target for various vulnerabilities, including application security breaches, unauthorized access, and replay attacks.

#### Network layer.

The network layer within the IoT architecture is responsible for data routing and ensuring secure transmission throughout the system. It facilitates data communication through various protocols, such as Zigbee and 6LoWPAN [14], and is heavily dependent on the middleware layer for subsequent processing and operations. The network layer also plays a crucial role in managing the connectivity of IoT devices, ensuring that data is transmitted efficiently and securely.

### IoT security models

The versatility of IoT is truly remarkable, with its applications spanning from healthcare to logistics, smart cities, infrastructure monitoring, e-governance, smart car parking, rescue and emergency support centers, and more. This wide range of uses not only underscores the increasing importance of IoT but also presents a fascinating landscape of possibilities. The transmission of data via wireless channels and its security are major concerns, and to address these, numerous organizations, researchers, and businesses have presented various models [15–19] to secure the data breaches noted in IoT. Let’s delve into some of these models:

#### Risk-based model.

This model offers a unique advantage by using artificial intelligence (AI), deep learning, and risk assessment algorithms to identify threats in IoT. It can also generate automated responses when data breaches occur, providing a swift and effective countermeasure [15].

#### Zero-trust security model.

The Zero-Trust Security Model, a robust system that protects sensitive information, holds significant promise for IoT. Its wide adoption in private networks is a testament to its potential, especially in light of the inadequacy of existing models in meeting the security requirements for IoT [16].

#### Machine Learning (ML) model.

The analyzed data, ML, has been used to develop algorithms that efficiently learn and adapt new risk assessment mechanisms. This model integrates ML with advanced encryption standards to build a hybrid system for IoT data security [17].

#### Blockchain-based model.

This is the newer technology adopted for IoT to provide better security, confidentiality, privacy, and integrity of exchanged information. Blockchain-based security models enhance transparency in analyzing the data received from IoT, self-executing capabilities, and decreasing the risk of unauthorized access [18].

#### Multi-factor authentication model.

The Multi-factor Authentication Model, a comprehensive system that uses passwords, hardware, biometrics, and unique identities, offers not only faster services but also robust security in IoT [19]. Its robustness provides a sense of reassurance in the face of IoT’s security challenges.

In this context, Nakkar et al. [20] introduced a multi-factor authentication model for the edge computing paradigm, emphasizing the potential of edge computing as a beacon of hope in the decentralized network topologies of the IoT era. Despite its security challenges, primarily due to the inherent wireless communication channel in the edge IoT structure, which introduces numerous security risks, the model based on symmetric cryptography offers a ray of hope. This model claims to provide robust security features like forward/backward secrecy, anonymity, and mutual authentication, instilling a strong sense of security and confidence in the potential of edge computing. Liu et al. [21] also proposed an adaptive and efficient multi-factor authentication model for edge-IoT environments that utilizes a simple hash-cryptographic algorithm to secure resource-constrained IoT applications. Jan et al. [22] also presented the same model for the dew computing paradigm using the ECC technique, and Zhang et al. [23] also presented a multi-factor model for an edge–fog–cloud architecture, enhancing security through a stateless mechanism.

#### Elliptic Cure Cryptography (ECC).

The algebraic structure of elliptic curves over finite fields is the foundation for the modern public-key encryption method called Elliptic Curve Cryptography (ECC). Compared to traditional cryptographic techniques such as RSA, ECC provides strong security with considerably smaller key sizes, making it a preferred choice for secure data transmission, encryption, and digital signatures, used by [21].

ECC is represented by the mathematical equation *y*^*2*^* = x*^*3*^* + ax + b*, where *a* and *b* are constants. An interesting property of this curve is that any straight line that intersects it twice will also intersect it a third time. This point can be reflected across the x-axis to create a new curve point. In this process, a randomly selected integer acts as the private key, while a pre-defined base point (known as the generator point) on the elliptic curve is multiplied by the private key to generate the corresponding public key adopted by [23]. Although this computation is straightforward, reversing it is considerably difficult.

The robust security offered by ECC is a significant factor in its widespread adoption across contemporary cybersecurity applications, including IoT, cloud computing, edge computing, fog computing, and dew computing. ECC stands out as a powerful and efficient cryptographic technique, delivering strong security with minimal resource consumption, thus instilling in users a sense of safety and protection.

### IoT security requirements

Security is still a major concern for IoT. The following are the key requirements for developing a security model/scheme for exchanging information from IoT systems into edge servers over a wireless channel [24–26].

#### Confidentiality.

Data secrecy is not just a desirable feature, but an essential one to prevent data from being disclosed. Many low-resource IoT applications communicate sensitive information, and an attacker can intercept and hack valuable information when transmitted from IoT towards the edge server. This eavesdropping may cause catastrophic harm to the edge-IoT ecosystem because the adversary can use the information and user for several malicious activities. The potential risks of not ensuring confidentiality are significant and should be a primary concern in IoT security [24]. ECC in combination with SHA256 can guarantee this security feature in the proposed protocol.

#### Integrity.

Integrity is not just a security feature, but a requirement for the information communicated over an insecure channel. Without ensuring integrity, an attacker may change the content of messages sent from IoT to the edge server or the intended recipient. The potential for catastrophic harm in the absence of integrity is significant, making it a critical aspect of IoT security [25].

#### Availability.

Availability is a critical aspect of IoT security, particularly in ensuring continuous access to data. IoT’s efficient operation relies on monitoring physical environments, gathering information, storing and analyzing data, and transmitting it to the edge server over wireless communication channels. If this security feature is not ensured, an attacker could compromise the gathered sensitive information, potentially leading to catastrophic effects in life-saving applications due to delays or unavailability of essential data [26].

#### Authentication.

Authentication can be either a message or device authentication. It is a critical component of all security models. Without proper authentication, no one guarantees security, privacy, and all associated security features because it allows the verification of a reliable end device while transmitting data to the edge server [25,26].

## Related works

The edge-IoT ecosystem faces several critical security issues and challenges impacting its optimum performance and integration. These challenges, which stem from the complexity of managing diverse IoT, ensuring security, and maintaining efficient data processing, are urgent and require immediate attention [27,28]. The heterogeneity and resource-constrained nature of edge-connected IoT exhibit significant diversity in hardware and software, complicating application deployment and management across multiple sites. These resource-constrained devices need more computational power for complex analytics, necessitating innovative resource management strategies [29]. However, security is still a major concern due to the decentralized and distributed nature of the edge computing paradigm that easily introduces new security vulnerabilities and can only be managed by requiring robust measures for protecting sensitive data [30]. Privacy, scalability, and interoperability issues arise when the data is transferred from IoT to the edge server over a wireless channel. The task-offloading algorithm and effective security protocol can handle such problems by integrating numerous heterogeneous IoT devices with edge servers [31]. Ensuring scalability, privacy, security, and flexibility challenges for the edge IoT ecosystem can significantly present opportunities for innovation in edge computing technologies, leading to more resilient and efficient IoT ecosystems [32,33]. Addressing these issues will be essential for developing smart applications and services for such a decentralized environment [34].

The IoT network is rapidly expanding, with billions of low-cost devices connecting, which poses a potential security threat. With their limited energy, storage, and computation capabilities, these devices are increasingly vulnerable as they access the open network. It is important to note that these limitations often make protecting those using traditional cryptographic techniques impossible. Consequently, numerous researchers have proposed various innovative security mechanisms to protect these transmissions. For instance, the research by Dubrova et al. [35] examined whether the restrictions of resource-constrained IoT devices are met by the Cipher-based Message Authentication Code (CMAC) and KECCAK Message Authentication Code (KMAC). They [35] further demonstrated that CMAC is smaller and more potent than KMAC, providing robust security while using minimal resources. The researchers from [36,37] argued that public key cryptography required a large percentage of processing overhead; existing techniques based on public key cryptography still need to be improved for resource-constrained IoT applications. Therefore, they [36,37] proposed innovative security mechanisms for substantial computational complexity, robust authentication, data security, and the preservation of privacy using group management techniques, which could significantly enhance the security of the IoT network.

Similarly, the researcher in [38] suggested a certificate-based method for secure authentication across the edge-IoT ecosystem. Their [38] scenario offered a mutual authentication among the wearables/sensors embedded in the patient’s bodies and confirmed the confidentiality, and data leakage did not occur while using their scheme. They [38] also presented a low-complexity security and authentication system to reduce energy consumption and increase the lifespan of the medical system because each IoT device has a limited battery capacity. The researchers of [39] presented an implementation scenario for a hospital in the healthcare platform using ZigBee mesh technology, a practical and efficient solution. In contrast, the researchers in [40] demonstrated that numerous wireless media are available in clinical settings, including one with a particular purpose related to m-health and e-health. They proposed a reassuring solution using NFC for the patient data of type m-health status, arguing that information would be gathered from that exact room using Bluetooth technology and onward be transmitted to the edge node in a secure method; they [40] proposed IPv6 over Low-Power Wireless Personal Area Networks (6LowPAN) for the data of type e-health by monitoring each unit of the healthcare industry.

Yenuganti et al. [41] used accelerometer data from sensors to establish sensor authentication, checking whether the devices were being used by the same person’s body around the waist. They [41] stated that accelerometer data, which measures the body’s movement, can easily be used to analyze a person’s walking patterns. The results they [41] obtained from the method based on these walking patterns, collected through a cellphone with accelerometers placed in the exact location in the person’s body, revealed that these patterns are similar. In [42], the symmetric and asymmetric security technique for IoT was discussed in detail, providing practical insights for implementation. This practical guidance is particularly valuable for professionals in the field. The researchers in [43,44] have discussed using a global hash function for defining the HMAC. The algorithms they [42–44] have discussed include SHA-1, MD5, RIPEMD, and several HMAC variants like HMAC-SHA1, HMAC-MD5, and HMAC-RIPEMD.

The authors in [45] proposed a key agreement scheme, [46] presented a privacy-preserving security mechanism, and [47] designed an ECC-based scheme for resource-constrained IoT applications. They demonstrated that blockchain technology, a pioneering solution, is an efficient and secure message authentication solution. This technology can easily authenticate the edge-IoT ecosystem by controlling unauthorized user data and users’ transmitting security over the decentralized network. They [45–47] further highlighted the benefits of blockchain operations by thoroughly examining, revealing, and formally validating their mechanisms. Their [45–47] implementation approaches have achieved all the security goals defined and said that the ECC usage for authentication, compared to the other methods, provides an unparalleled level of security, instilling a sense of confidence in the data transmission from IoT. However, managing them requires a lot of computing and storage resources. They [48] have compared the CRC and KECCAK in the same environments, along with a combination of MD5 and SHA-1. After that, in [49], the MD5 and SHA-1 were used to design the IBOOS scheme. Radiofrequency (RF) is used to regulate software-defined networks (SDN) by providing error control and secure authentication [50]. A power-efficient solution was proposed in shipping identification and storage management using back-scatter technology [51] and tagged data transmitted through regulated sources. They [51] have simulated via MatLab by showing that their proposed scheme is efficient performance, offering detailed benefits of the efficient performance.

Tan et al. [52] highlighted the challenges to the industrial Internet of Things (IIoT) by arguing that the transformation of data in a conventional manner is not safe and is very slow; the IIoT collects, processes, stores, and transmits data among various machines for mechanical activity. However, collecting and transmitting IIoT data can be done through the open network, which can be affected when an unauthorized user can participate in the network. To eliminate the harmful activity of an attacker in the IIoT network, authentication is the most attractive technique that can be fulfilled through a digital signature [52]. For the authentication of users in the IIoT ecosystem, Karati et al. [53] proposed a certificateless signature-based scheme. They claimed that [53] is efficient regarding computation and communication overheads and safe against type 1 and 2 adversaries. Type 1 adversaries are those who can intercept and modify the communication between legitimate users, while type 2 adversaries are those who can impersonate a legitimate user. However, according to the detailed cryptanalysis of Karati et al. [53], Zhang et al. [54], and Zhang et al. [55], these schemes are not secure from type 1 and type 2 adversaries. Zhang et al. [55] also presented a certificateless signature-based scheme utilizing ECC and claimed that their scheme requires less computational effort. Xiong et al. [56] have developed a certificate-based approach for the IIoT environment, leveraging key insulation utilizing ECC. Rezaeibagha et al. [57] have introduced an enhanced approach using the same certificateless signature-based scheme that showed resilience to both type 1 and 2 adversaries. However, their scheme, like many others, is unsafe against forgery attacks, which can severely impact IIoT security.

Finally, Ali et al. [58] devised a certificateless approach for the IIoT environment, employing hyper-elliptic curve cryptography. They argued that their scheme is more efficient regarding computational and communication costs than the existing certificate-based signature schemes for the IIoT environment. However, their [58] scheme has these flaws, including the use of certificateless cryptography, which can affect the partial private key distribution algorithm and fails to provide the facilities for data aggregations. Verma et al. [59] present a significant advancement with their new approach that removes the partial private key distribution problem using certificate-based cryptography and includes the data aggregation technique that performs aggregation on data in run time. However, their [59] approach cannot withstand type 1 and type 2 adversaries and concerns unforgeability property [59]. Then, utilizing the bilinear pairing operation with the key insulation method, the authors in [60] used the certificate-based aggregate signature scheme. However, due to heavy operations in bilinear pairing, their scheme is not efficient and effective in performance. Hwang and Lee [61] proposed a certificate-based aggregate signature scheme using the elliptic curve point multiplication operations with a key insulation method.

In conclusion to the prolonged literature survey, it was crystal clear that despite the many advantages of cloud computing—including flexibility, accessibility, efficiency, and cost savings—it still faces challenges such as potential data loss, security issues, limited control, and availability concerns. To address these challenges, experts have introduced the edge computing paradigm, which aims to outperform cloud computing in these areas. Edge computing is directly connected to the Internet of Things (IoT), sensors, and wearable devices in a decentralized manner, distributing processing power closer to the data source rather than relying on a central cloud server for all computations. This approach enables faster data processing and reduced latency by handling data locally at the network’s edge, where it is generated. However, the resource-constrained nature of IoT devices, sensors, and wearables has led to numerous data breaches due to vulnerabilities associated with sensitive data proximity, physical tampering, privacy concerns stemming from user proximity to data collection, and the challenges of managing security across a multitude of edge devices. Current authentication schemes have not adequately addressed the security requirements of the edge computing paradigm; they often have design flaws, are vulnerable to various threats—such as impersonation, insider attacks, denial of service (DoS), and replay attacks—or suffer from inadequate performance due to reliance on resource-intensive cryptographic algorithms, such as modular exponentiations. Given the urgent need for robust security mechanisms in the dynamic and vulnerable edge IoT ecosystem, this article proposes an ECC-based robust authentication scheme tailored for resource-constrained IoT environments.

## System models

This section of the article presents the proposed network model and potential threats to the system. It outlines the objectives for designing a robust authentication protocol tailored for the edge-IoT ecosystem. The following sections will explain these fundamental concepts one by one:

### Network model

**Fig 1:**
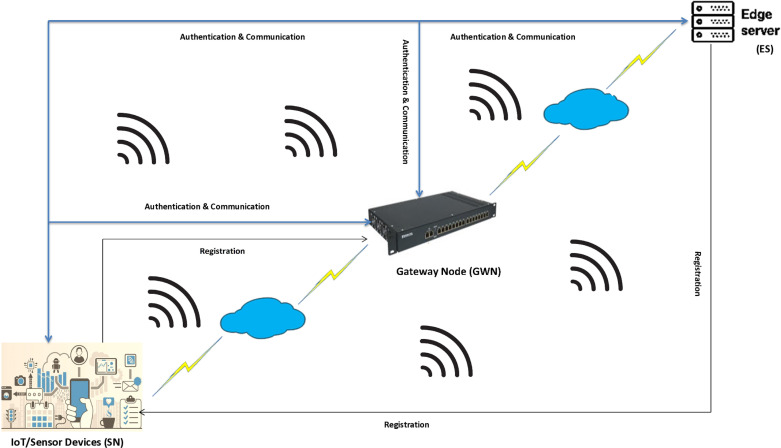
Network model.

The proposed network model is a robust system that powerfully underscores the security challenges of the edge-IoT environment, which is not only resource-constrained but also dynamic and vulnerable to potential threats. The proposed network model consisted of mainly three essential entities: the sensor/IoT device, the gateway node, and the edge server. The gateway node plays a critical role by facilitating networking and communication with a strengthened focus on security. Once registered with the gateway node, the IoT/sensor devices and edge server can be activated to execute various practical and tactical tasks, enhancing the practicality of the network model. This focus on security generates a network model suitable for multiple applications, including logistics, transportation, smart cities, car parking, and infrastructure surveillance, e-healthcare, and pipeline inspection. A diagrammatic representation of the network model is depicted in Fig 1. The functions of the participants in the proposed network model are detailed as follows:

#### Edge server.

The edge server, functioning similarly to a traditional server, is placed in individualistic locations. This strategic placement allows data processing close to the end user, particularly in environments like self-driving cars, smart homes, industrial settings (IIoT), hospitals powered by IoT technologies, search and resecure operations and support logistics. An edge server enhances responsiveness, enabling real-time solutions for processing and storing data from multiple IoT devices within the respective application areas while facilitating multi-tenant virtualization. However, this proximity introduces significant security risks, as adversaries may compromise system operations by infiltrating these processing units from both internal and external sources. Addressing this critical concern, this article will soon present a promising solution that is aimed at securing this emerging paradigm. This solution is a key part of the article and will be discussed in detail in the upcoming section.

#### Gateway.

The centralized entity/participant, a trusted contributor to the proposed Network model, facilitates edge IoT services for its clients. The employing approach generates parameters/credentials and executes secret keys stored in the memories of the participating entities for later establishing a mutual authentication and cross-synchronization of the ecosystem. The reliability of this entity is not just a feature but a foundational element of the system’s security, underscoring its importance and impact on the overall system. It offers networking capabilities to the entire system, registers each participant, and deploys them for effective task execution.

#### IoT/sensing devices.

The term “Internet of Things” refers to a category of objects capable of communicating with other devices and systems via the Internet or other communication networks. These devices, which are equipped with sensors, processors, software, and various technologies, have considerable potential to transform how we interact with technology and our surrounding environment. In the proposed network model, IoT/sensing devices will be placed in the application area for real-time data collection, should be sent to the gateway through a wireless channel, and from there, will be securely forwarded to the edge server for purposes, ensuring the utmost confidence in data transmission.

### Threat model

This research utilizes the threat models established by Canetti-Krawczyk (CK) [62] and Dolev-Yao (DY) [63]. According to these models, a system that offers online services may encounter a variety of threats, including the following:

#### False data injection threat.

Under the False Data Injection Threat, an adversary could manipulate the information collected by sensors or wearable devices, potentially capturing sensitive credentials for malicious use. The potential damage of such an attack underscores the urgency of robust security measures.

#### Privacy threat.

An adversary could exploit an open network channel by employing identity theft techniques, redirecting packet flows, and de-authenticating the involved entities [64]. Such actions would jeopardize the privacy of the end user and other system participants.

#### Traffic analysis threat.

An adversary may intercept packets exchanged among participants, analyze their content, and exploit this information maliciously. Since data collection occurs through sensors or wearables utilizing wireless communication, this medium presents vulnerabilities that can be exploited. If the security mechanisms are inadequate, an adversary can easily scrutinize the transmitted credentials and messages, potentially extracting sensitive internal information from these packets.

#### Access control threat.

In the case of an Access Control Threat, an adversary can uncover policies, rules, and the legitimacy of authorized participants, subsequently gaining unauthorized control. This threat underscores the necessity for robust security measures, as proactive strategies are essential to mitigate such risks.

#### Identity spoofing threat.

An adversary gaining access through a spoofed identity can impersonate a legitimate user or a system component. This allows the adversary to gain unauthorized access or potentially disrupt normal operations.

#### Replay attack.

In a Replay Attack, an attacker can capture data from an open channel, eavesdrop on communications, and later utilize this information for illegal system access. This threat underscores the importance of continuous monitoring and vigilance, as security measures should be persistent rather than one-off efforts.

#### Desynchronization threat.

When a legitimate user attempts to update their identity for an upcoming session, they may remain unaware of changes to their user identity, leading to a desynchronization threat. This can result in the user being unable to connect with the legitimate server for services, significantly disrupting their experience. Furthermore, if an adversary gains access to the server and disrupts shared memory synchronization, the desynchronization threat is exacerbated.

#### Man-in-the-middle (MITM) attack.

In this scenario, an adversary intercepts packets transmitted to the server, reroutes them, modifies the communication flow, and steals the identity of the original sender, thereby impersonating the entire system. The stealth and complexity of this attack make it crucial for us to have advanced detection and prevention measures in place.

#### Stolen-verifier attack.

Here, an attacker steals a sensor, wearable device, or IoT device and extracts its internal secret credentials. These stolen credentials can be misused for potential replay, masquerade, or denial-of-service (DoS) attacks, highlighting the severity of the situation.

#### Analytical attack.

An adversary employs cryptanalysis, power analysis, and reverse engineering techniques to derive valuable parameters from exchanged messages. This enables them to quickly recover cryptographic session keys, which they can then exploit to establish their own session with the system.

### Design goals

When designing a security protocol, several key features and objectives must be achieved, including mutual authentication, anonymity, confidentiality of identities, unforgeability of messages, forward secrecy, mitigation of secret key leakage, and identity authentication [62–64]. The primary design goals for the proposed security mechanism are as follows:

#### Mutual authentication.

This fundamental aspect of network security ensures that all participating entities authenticate one another through a communication channel. This process not only guarantees that network users are interacting with genuine entities but also provides a sense of security to servers, assuring them that all sensors, wearables, or IoT devices seeking access are doing so for authorized purposes.

#### Anonymity.

Safeguarding the identity of sensors, wearables, or IoT devices over a public channel is crucial in the design of the security scheme. There must be no means to link an individual’s actions to a valid identity within an edge server or gateway node that claims to maintain anonymity. While achieving anonymity is an essential design goal in cryptographic systems, it comes with challenges; the system’s overall security may be compromised if not implemented correctly.

#### Confidentiality.

The data generated by sensors, wearables, or IoT devices must be kept confidential and accessible solely to the designated edge server or legitimate recipient. The ciphertext should remain unrecognizable to an attacker on the public network and should not resemble the plaintext in any way. Only the edge server or authorized IoT device should be able to decrypt the ciphertext and retrieve the original plaintext to ensure confidentiality.

#### Unforgeability.

If a forgery can generate a valid message from an open network channel that successfully passes through the hash function, then the protocol fails to ensure unforgeability. However, the proposed scheme effectively mitigates this risk.

#### Forward secrecy.

Perfect Forward Secrecy (PFS), often referred to as Forward Secrecy (FS), is a protocol feature that continuously changes the keys for encrypting and decrypting data. This ongoing process ensures that even if the most recent key is compromised, only a limited amount of sensitive data is exposed, instilling confidence in the system’s resilience and preventing an adversary from accessing other credentials in the system.

#### Secret key leakage.

It occurs when the single key necessary for encrypting and decrypting communications is a shared secret between the sender and recipient.

#### Identity authentication.

Authentication is an essential component of network security that validates individuals’ identities. It relies on additional information that is only accessible to a specific person. By incorporating an extra layer of identity verification, authentication not only validates the identity but also expands the range of identifying information required to achieve a valid match, ensuring the thoroughness of the security measures.

## Proposed authentication scheme

This section of the research work continues the discussion by illustrating the proposed solution for the system architecture presented in section 4 of the article. The proposed solution consists of sequential stages, including edge server registration, user registration, and mutual authentication. These phases of the proposed protocol are explained separately, and Table 1 displays the terminology for the different notations used for designing the protocol.

**Table 1:** Notations used and their descriptions.

Notation	Meaning	Notation	Meaning
*GWN*	Gateway Node	*R*_*SN*_	Random Number for IoT/Sensor Node
*S*_*ES*_	Edge Server	*U*_*HC*_	End-User
*SN*	Sensor Node/IoT	*R*_*ES*_	Random Number for Edge Server
*R*_*GWN*_	Random number of Gateway Node	*⊕*	Exclusive OR Operation (Bitwise)
*ID*_*SN*_	Sensor Node/IoT/User Identity	*E*_*P*_ *(x, y)*	Point x, y over a finite field F_P_
*PW*_*SN*_	Sensor Node/IoT Password	*B*_*SN*_	Sensor Node/IoT/User Biometrics
*S*_*GWN*_	Gateway Node Secret Number	*H(.)*	Bio-Hash Function
*||*	Concatenation Function	*h()*	One-Way hash function
*(+)*	Point Addition	*(×)*	Point Multiplication
*ID*_*ES*_	Edge Server Identity	*r*_*1*_ *, r*_*2*_ *, r*_*3*_	Random numbers

### Setup phase

In this protocol phase, the gateway node (GWN) meticulously selects a curve E_P_(x, y) over a finite field F_P_ and chooses a point α on a curve. The gateway node (GWN) then determines an integer number S_GWN_, called a secret key and calculates PK_α_=S_GWN_×α with utmost precision as the public key. Further, the gateway node (GWN) selects two points (x, y) as secret numbers. The secret keys with the GWN are {x, y, S_GWN_}, and the public keys are {PK_α_, α, E_P_}. These keys are then securely shared through a public network channel to all the participating entities, ensuring that attackers can’t pick this public key at any stage for potential attacks.

### Edge server registration phase

This phase is accomplished in the following steps:

**Step 1**: First, the operator selects a unique identity ID_ES_ for the edge server and sends it {ID_ES_} to the gateway node (GWN) over a secure channel.

**Step 2**: The gateway node (GWN), upon receiving the {ID_ES_} message, checks {ID_ES_} in its reliable and secure database. If found in the record of gateway, it advises the operator to choose another identity for the edge server (ES), for not matching the identity the GWN computes K_GWN_ = h(ID_ES_||x||y) and sends it back to the edge server (ES) over the same private channel.

**Fig 2:**
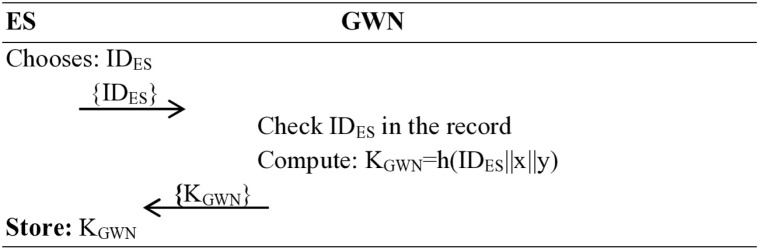
Edge server registration phase.

**Step 3**: Upon receiving {K_GWN_} message from the GWN, the edge server (ES) stores it in its memory for future correspondence, specifically, the computed key {K_GWN_}, as shown in Fig 2.

### Sensing/IoT device registration phase

The sensor wearable or IoT registration, or simply user registration, is the crucial phase of the proposed protocol. This highly technical process involves a series of complex steps, demonstrating the sophistication of the system.

Step 1: The legitimate user operating the senor node/IoT device can provide a unique identity ID_SN_, imprint biometrics (B_SN_), and a unique password (PW_SN_) through the application installed for their access. Upon entering these credentials, the device randomly picks a number r_1_, computes HPW_SN_ = h(PW_SN_||ID_SN_||r_1_), A_SN_ = H(B_SN_||r_1_), and transmits {ID_SN_, HPW_SN_, A_SN_} message towards the gateway node (GWN) over a secure path, ensuring the highest level of security in the process.

Step 2: The gateway node (GWN), when receiving {ID_SN_, HPW_SN_, A_SN_} message, confirms whether the sensor node is already registered or a new one; if the record matches with the stored record, a message will display to tell the operator for registering another IoT/sensing device which is not previously registered, if not, it computes HID_SN_ = h(ID_SN_||x||y), C_SN_ = HID_SN_ ⊕ h(HPW_SN_||A_SN_), D_SN_ = h(ID_SN_||HPW_SN_||A_SN_), G_SN1_ = h(ID_SN_||x), and G_SN2_ = h(ID_SN_||y). The gateway node (GWN) chooses two points in the curve, Z_1_=(G_SN1_, G_SN2_) and Z_2_=(HID_SN_, HPW_SN_), and calculates the sandwich (middle) points between Z_1_ and Z_2_ denoted by (uv_1_, uv_2_), which is obtained through uv_1_=(Z_1_ + HID_SN_)/2 and uv_2_=(Z_2_ + HPW_SN_)/2. The gateway node (GWN) computes I_1_ = h(HID_SN_||HPW_SN_) × α, I_2_=(uv_1_, uv_2_)+h(HID_SN_||HPW_SN_) ×PK_α_) and stores {C_SN_, D_SN_, I_1_, I_2_, h(.), H(.)} in the memory of gateway and sends {C_SN_, D_SN_, I_1_, I_2_, h(.), H(.)} message towards the user side senor node/IoT via a secure channel.

**Fig 3:**
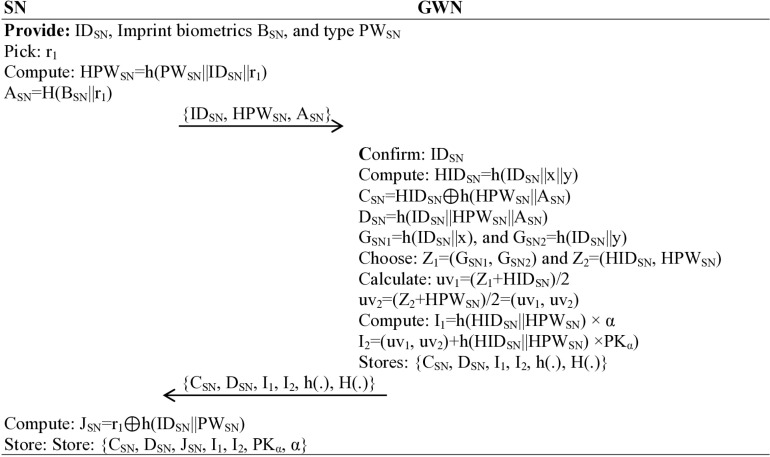
SN/IoT or user registration phase.

Step 3: The user/sensor node/IoT, when receiving {C_SN_, D_SN_, I_1_, I_2_, h(.), H(.)} message, plays a crucial role in completing the registration process. By computing J_SN_ = r_1_ ⊕ h(ID_SN_||PW_SN_) and storing the necessary data, as shown in Fig 3.

### Mutual authentication phase

All three participants must agree on a single session key, which is then used for secure communication. Such mutual authentication of all the participants requires five (5) steps described as follows:

**Step 1**: The operator provides a legal identity (ID_SN_) issued to them by the authorities, a password (PW_SN_) that they have set for themselves, and imprints biometrics (B_SN_) that were already generated to the system during the registration phase, and computes r_1_ = J_SN_ ⊕ h(ID_SN_||PW_SN_), A_SN_ = h(B_SN_||r_1_), HPW_SN_ = h(ID_SN_||PW_SN_||r_1_)

D_SN_^*^ = h(ID_SN_||HPW_SN_||A_SN_), confirms D_SN_? = D_SN_^*^; if it doesn’t match, the system discarded and considers a potential reply attack; otherwise, the system computes HID_SN_ = C_SN_ ⊕ h(HPW_SN_||A_SN_), extracted a nonce N_SN_, and note the time T, calculates E_1_ = N_SN_×α, E_2_=(HID_SN_, HPW_SN_)+N_SN_ × PK_α_, E_3_ = N_SN_ ⊕ h(HPW_SN_||uv_1_||I_1_), E_4_ = h(HID_SN_||N_SN_||uv_2_||I_2_||T), and sends {E_1_, E_2_, E_3_, E_4_, T} message towards the gateway node (GWN), a critical component, over an open channel.

**Step 2:** The gateway node (GWN), upon receiving {E_1_, E_2_, E_3_, E_4_, T} message, verifies the timestamp through a matching algorithm by calculating the received timestamp from the current time T_c_-T≤∆T if it doesn’t validate for the pre-defined time-threshold, should be considered an outdated message and a potential man-in-the-middle attack, the process is terminated and denies message is displayed. While for successful verification of the time, the GWN recovers ID_SN_, HPW_SN_ from E_2_ utilizing the private key of gateway d_GWN_ (ID_SN_, HPW_SN_)=E_2_-d_GWN_ × E_1_, calculates HID_SN_ = h(ID_SN_||x||y), I_1_ = h(ID_SN_||x), I_2_ = h(ID_SN_||y), E_1_=(uv_1_, uv_2_)=((HID_SN_ + I_1_)/2, (HPW_SN_ + I_2_)/2), N_SN_ = E_3_ ⊕ h(HPW_SN_||uv_1_||I_1_), E_4_^*^ = h(HID_SN_||N_SN_||uv_2_||I_2_||T), and verifies E_4_^*^? = E_4_, if it doesn’t verify, again the process stops and terminated for potential MIT, DoS or replay attacks, however, if found valid, the gateway node (GWN) generates a nonce N_GWN_ and note the present time T, calculates E_5_ = K_GWN_×α, E_6_=(I_1_, I_2_)+K_GWN_ × PK_α_, E_7_=(uv_1_, uv_2_)+K_GWN_ × PK_α_, E_8_=(N_GWN_||N_SN_)⊕h(HID_SN_||HPW_SN_||T), E_9_ = h(N_GWN_||N_SN_||T||I_1_||uv_1_||HPW_SN_), and transmits {E_5_, E_6_, E_7_, E_8_, E_9_, T} message to the edge server (ES) over an insecure (wireless) channel.

**Step 3**: The edge server (ES), upon receiving {E_5_, E_6_, E_7_, E_8_, E_9_, T} message, again verifies the timestamp through a matching algorithm by calculating the received timestamp from the current time T_c_-T≤∆T; if it doesn’t verify, the ES considered the received message an outdated one, the process become terminated and deny message will be displayed, however, for successful verification of the time, the edge server (ES) calculates (I_1_, I_2_)=E_6_-K_GWN_ × E_5_, (uv_1_, uv_2_)=E_7_-K_GWN_ × E_5_, HID_SN_ = 2 × uv_1_–I_1_, HPW_SN_ = 2 × uv_2_–I_2_, (N_GWN_||N_SN_)=E_8_ ⊕ h(HID_SN_||HPW_SN_||T), E_9_^*^ = h(N_GWN_||N_SN_||T||I_1_||uv_1_||HPW_SN_), confirms E_9_^*^? = E_9_, if doesn’t confirm, the process terminated, the message discarded and deny message displayed, however, for successful validation of E9*? = E9, the edge server (ES) also generates a nonce N_ES_, records the present timestamp T, calculates E_10_=(N_ES_||N_GWN_)⊕h(HPW_SN_||N_SN_), E_11_=((HID_SN_ + uv_1_)/2, (HPW_SN_ + uv_2_)/2), L_2_=(HID_SN_ ⊕ T||HPW_HC_), C_SN_ = HID_SN_ ⊕ h(HPW_SN_||A_SN_) and sends {E_10_, E_11_, L_2_, C_SN_, T} message back to the Gateway node GWN over a wireless channel.

**Step 4**: The GWN, when receiving E_10_, E_11_, L_2_, C_SN_, T} message, again verify the timestamp through a matching algorithm by calculating the received timestamp from the current time T_c_-T≤∆T if it doesn’t qualify for the pre-defined time-threshold, the GWN considered it a potential MITM attack, the process is terminated and denies message displayed, however, for successful verification of the timestamp, the GWN calculates HID_SN_ = 2 × uv_1_-I_1_, HPW_SN_ = 2 × uv_2_–I_2_, C_SN_ = HID_SN_ ⊕ h(HPW_SN_||A_SN_), L_2_^*^=(HID_SN_ ⊕ T||HPW_SN_), again verifies L_2_^*^? = L_2_; if doesn’t, discarded and terminate the process, but for successful validity, the GWN note the present time T and transmits {E_10_, β, T} message to the user/senor/IoT/mobile-device over a public channel.

**Fig 4:**
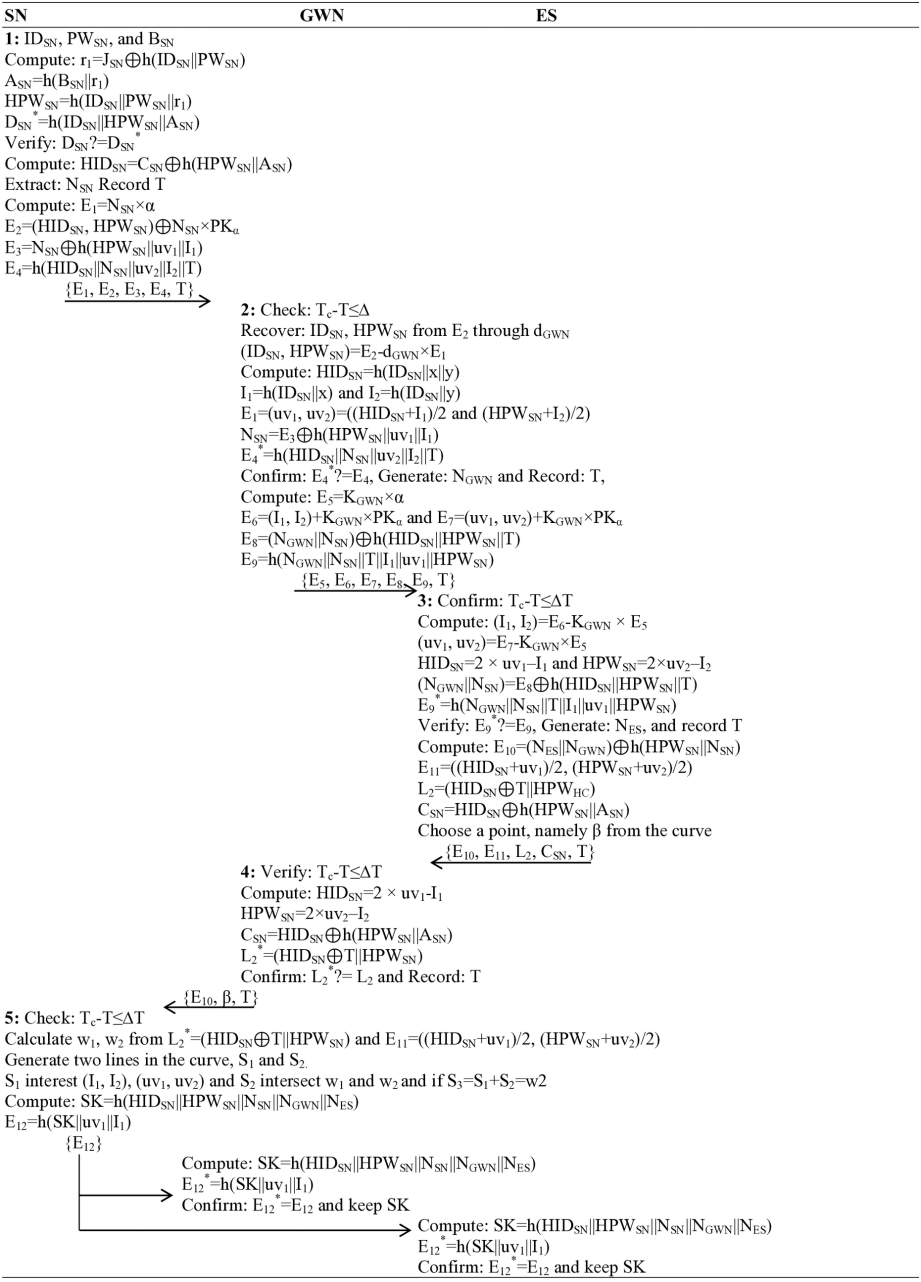
Mutual authentication phase.

**Step 5:** The user/senor/IoT/mobile device, upon receiving {E_10_, β, T} message, is tasked with a complex process. They verify the timestamp through a matching algorithm by calculating the received timestamp from the current time T_c_-T≤∆T. If the verification fails, the process is terminated. However, for successful verification of the timestamp, the user side also confirms w_1_, w_2_ from L_2_^*^=(HID_SN_ ⊕ T||HPW_SN_) and E_11_=((HID_SN_ + uv_1_)/2, (HPW_SN_ + uv_2_)/2), generates two lines in the curve, S_1_ and S_2_, where S_1_ intersect (I_1_, I_2_), (uv_1_, uv_2_) and S_2_ intersect w_1_ and w_2_. If the intersection of S_1_ and S_2_ is S_3_ and becomes equal to w2, it signifies that the participants are successfully matched. This process, with its intricate details, computes the session key SK = h(HID_SN_||HPW_SN_||N_SN_||N_GWN_||N_ES_) and E_12_ = h(SK||uv_1_||I_1_) and sends {E_12_} to both gateway node (GWN) and edge server (ES). There, they both note and calculate SK = h(HID_SN_||HPW_SN_||N_SN_||N_GWN_||N_ES_), E_12_^*^ = h(SK||uv_1_||I_1_), confirmed E_12_^*^ = E_12_ and keep SK as session secret key, as shown in Fig 4.

## Analysis and discussions

This section presents the security analysis formally through real or random (RoR) model [65] and informally through realistic discussion while the performance analysis of the proposed protocol can be tackled through measuring storage, communication and computation costs. These are explained one by one as follows:

### Formal security analysis

Suppose ƿ means the protocol/partnering/participants, Ʀ is the responder, i is the i^th^ instance of SN, j is the j^th^ instance of GWN, and k is the k^th^ instance of the ES. And let the adversary 𝓐 persistently run different queries on the oracle with the help of challenger ℂ, demonstrating their determination to break the protocol. The 𝓐 action while running the different queries is discussed as follows:

*a: Send(*$\prod_{p}^{i}M$*), Send(*$\prod_{p}^{j}M$*), and Send(*$\prod_{p}^{k}M$*):* In these instances, the challenger ℂ executes the different instances (i^th^, j^th^, and k^th^) along with a message M. The results obtained by ℂ are then shared with 𝓐.

*b: Hash(*$\prod_{p}^{i}M$*), Hash(*$\prod_{p}^{j}M$*), and Hash(*$\prod_{p}^{k}M$*):* The challenger ℂ plays a crucial role in maintaining a list of tuples, initially storing L_K_ as empty in its memory at the first stage, and later, (M, O). When ℂ retrieves a value from L_K_ and compares it with the list using the oracle, it obtains a value, says O, which it then shares with 𝓐.

*c: Login(*$\prod_{SN}^{i},\;p$*), Login(*$\prod_{GWN}^{j},\;p$*), Login(*$\prod_{ES}^{k},\;p$*):* This query is executed by the adversary 𝓐 by launching an eavesdropping attack on ƿ including SN, GWN and ES participants.

*d: Corrupt(*$\prod_{SN}^{i},\;p$*), Corrupt(*$\prod_{GWN}^{j},\;p$*), Corrupt(*$\prod_{ES}^{k},\;p$*):* 𝓐 launching a forgery attack on ƿ by stealing secrets from the memory of the GWN.

*e: Reveal(*$\prod_{SN}^{i}r)$*, Reveal(*$\prod_{GWN}^{j}r)$*, Reveal(*$\prod_{ES}^{k}r)$: 𝓐 disclosing the SK computed by ƿ containing SN, GWN and ES.

*f: Test(*$\prod_{SN}^{i}r)$*, Test(*$\prod_{GWN}^{j}r)$*, Test*$\prod_{ES}^{k}r)$*(:* In these tests, ℂ desires to check some specific parameters d_ES_ (secret key) called λ^∊^ in the SK and sends to 𝓐. The secret key is a crucial element in the protocol, and its compromise could lead to a breach of security. However, 𝓐 doesn’t know whether the secret key is authentic or not; for making the exact decision regarding the secret key, 𝓐 flips a coin; if the value they get is 1 means 𝓐 win the value ℂ return is d_ES_ (secret key), and if get 0, means 𝓐 doesn’t succeed for d_ES_ (secret value), but it is also a chance with 𝓐 not to get anything ⟘ means “null”.

*g: Semantic Security:* Suppose 𝓐 runs the *Test(.)* query on ƿ by polynomial times attempts *t*. According to the birthday paradox [66], multi-collision of hash queries includes q_E_ (execute query), q_S_ (send query), and q_H_ (hash query). If 𝓐 desires to launch a hash collision attack on it, then the advantage with 𝓐 for breaking ƿ in polynomial times attempts *t* is expressed mathematically as under:


$$\begin{array}{c} {ADV}_{𝒜}^{p}(t)\leq \frac{{\left(q_{S}+q_{E}\right)}^{2}}{n}+\frac{{\left(q_{H}\right)}^{2}}{2^{L_{h}}}+2{ADV}_{𝒜}^{Key}(t) \end{array}$$ (1)

Suppose L_h_ is a list of many of the same images of the hash function, and when 𝓐 obtained any output, it matches with the list while the average length of hash codes in bits is ${n=2}^{L_{h}}$. The adversary 𝓐 is now trying to break the semantic security of the ƿ in the following steps:

Step 1: In this step, 𝓐 launches an active attack on ƿ; to do so, 𝓐 has winning chances that can be expressed mathematically as follows:


$$\begin{array}{c} {ADV}_{𝒜}^{p}(t)\leq 2P\;\left[W_{𝒜}^{0}\right]-1 \end{array}$$ (2)

Step 2: In this step, 𝓐 hack ƿ and compute SK; the winning chances with 𝓐 in doing so can be expressed mathematically as follows:


$$\begin{array}{c} {ADV}_{𝒜}^{p}(t)\leq P\left[W_{𝒜}^{1}\right]-P\left[W_{𝒜}^{0}\right] \end{array}$$ (3)

Step 3: in this step, 𝓐 has the potential to try to crack the publically transmitted key of ƿ; the advantage with 𝓐 in winning the cracking key polynomial times attempt (t) can mathematically be represented as:


$$\begin{array}{c} P\left[W_{𝒜}^{2}\right]-P\left[W_{𝒜}^{1}\right]=Adv_{𝒜}^{Key}(t) \end{array}$$ (4)

Step 4: The 𝓐 launches a hash collision attack created in ƿ; the mathematical representation of the winning chances with 𝓐 in the hash collision attack is:


$$\begin{array}{c} P\left[W_{𝒜}^{3}\right]-P\left[W_{𝒜}^{2}\right]\leq \frac{(q_{S}+q_{E}{)}^{2}}{2n}+\frac{q_{H}^{2}}{2^{l_{h}+1}} \end{array}$$ (5)

and


$$\begin{array}{c} P\left[W_{𝒜}^{3}\right]=\frac{1}{2} \end{array}$$ (6)

From eq: (1–6), we get


$$\frac{1}{2}{ADV}_{𝒜}^{p}\leq \frac{(q_{S}+q_{E}{)}^{2}}{2n}+\frac{q_{H}^{2}}{2^{l_{h}+1}}+Adv_{𝒜}^{Key}(t)$$ (7)

$${DV}_{𝒜}^{p}\leq \frac{(q_{S}+q_{E}{)}^{2}}{n}+\frac{q_{H}^{2}}{2^{l_{h}}}+2Adv_{𝒜}^{SHA}(t)$$ (8)

### ProVerif validation

The verification process, which rigorously checks for correctness, robustness, and random numbers cross-verification, involved simulating the proposed protocol using the esteemed software verification toolkit ProVerif [67]. The summary of the results after execution demonstrated that the attacker couldn’t breach the session secret key SK at any stage. The result summary also confirms that the confidentiality and the secure exchange of the SK among all the participants are key highlights and correct. This secure exchange of the session key should instil confidence in the security and correctness of the proposed protocol and the secure cross-synchronization of random numbers among the participants, as shown below:

---------------------------------------------------------

Verification summary

Query inj-event(endSN(IDSN)) ==> injevent(startSN(IDSN)) is true.

Query inj-event(endGWN(IDGWN)) ==> inj-event(startGWN(IDGWN)) is true.

Query inj-event(endES(IDES)) ==> inj-event(startES(IDES)) is true.

Query not attacker(SK[]) is true.

---------------------------------------------------------

### Informal security analysis

The pragmatic discussions regarding well-known attacks for the proposed protocol are now presented in this section of the article as follows:

#### Resists replay attack.

Suppose 𝓐 is retransmitting an irrelevant message towards the system using the proposed protocol or tries to intercept the open channel interactively. We have set timestamp check T_c_-T≤∆T at each round trip of the protocol, which strongly prohibits the adversary from launching a replay attack. Also, the proposed protocol, fortified with a 160-bit long ECC key, 60-bit random numbers, secret keys, curve points, and unique identities, is designed to prevent such attacks. The message, a random number in ciphertext format, is critical in the computation and verification process, underscoring its importance. In this context, the authentication phase also consisted of D_SN_? = D_SN_^*^, E_5_ = K_GWN_×α, and L_2_^*^? = L_2_ checks, a key factor in ensuring an attacker cannot launch a replay attack. Therefore, the proposed protocol is resisting a replay attack.

#### Withstands impersonation attack.

The proposed protocol is designed to withstand impersonation attacks; if 𝓐 impersonates as a legal user, they have to generate exact biometrics B_SN_, password PW_SN_ and identity ID_SN_ and use the information in the transmitted message like ECC-key PK_α_, and random nonce N_SN_ to interaction the other participant which of course they cannot succeed.

#### Resists man-in-the-middle attack.

If 𝓐 intercepts the communication line and copies the first message {E_1_, E_2_, E_3_, E_4_, T} of the protocol, and 𝓐 desires to find out something useful using for later on showing their self as a legitimate user, they have to passes from several computation steps like E_1_ = N_SN_×α, E_2_=(HID_SN_, HPW_SN_)⊕N_SN_ × PK_α_, E_3_ = N_SN_ ⊕ h(HPW_SN_||uv_1_||I_1_), and E_4_ = h(HID_SN_||N_SN_||uv_2_||I_2_||T), which of course cannot calculate the hash code, long public key, and other credentials. Therefore, the proposed ECC-based authentication protocol is designed to resist man-in-the-middle attacks.

#### Resists traceability attack.

The proposed method ensures an attacker cannot follow any legal participant session due to interactive data, i.e., {E_5_, E_6_, E_7_, E_8_, E_9_, T} sent between the GWN and the edge-server. Suppose 𝓐 tries to trace out the sensor, wearables or any IoT device. In that case, 𝓐 has to pass from these computation steps r_1_ = J_SN_ ⊕ h(ID_SN_||PW_SN_), A_SN_ = h(B_SN_||r_1_), HPW_SN_ = h(ID_SN_||PW_SN_||r_1_), D_SN_^*^ = h(ID_SN_||HPW_SN_||A_SN_) and HID_SN_ = C_SN_ ⊕ h(HPW_SN_||A_SN_) which is not possible for 𝓐 to do, because the messages communicated are built from numerous credentials utilize distinct random integers for every new session. The authorized IoT uses r_1_, N_SN_ and PK_α_, and the gateway uses r_2_, N_GWN_ and d_GWN_ to update all the associated parameters like identities, password and biometrics that ensure the utilization of numerous credentials for SK computation for different sessions. Ultimately, the IoT/sensor devices hide the sensor’s identity during many visits following a single authentication using the gateway’s identification and the current timestamp. According to the communication messages, 𝓐 cannot track down any participant in the proposed protocol.

#### Offers perfect forward & backward secrecy.

Even if 𝓐 knows the system’s long-term shared key, still 𝓐 cannot calculate the session key because SK is fully dependent on ECC key, identities, and random numbers, which is different in each session. The 𝓐 faces complications because of the requirement for secret shares to calculate SK; however, generating the new random numbers for every session adds another layer of security, making it difficult to decipher the session key SK of earlier or later sessions. 𝓐 only hope to determine the current session key or recreate the prior ones, which requires high proficiency in messaging.

### Comparative analysis (Security Functionalities)

When comparing the proposed protocol with state-of-the-art schemes regarding security functionalities, the results demonstrate that the proposed protocol offers comprehensive security coverage. It resists all known threats and incorporates the mentioned security functionalities, as shown in Table 2.

**Table 2:** Comparative Analysis (Security Functionalities).

Schemes → Security Functionalities↓	Alghamdi et al. [68]	Algarni et al. [69]	Jan et al. [70]	Irshad et al. [71]	Ghani et al. [72]	Proposed
Resists Replay Attack	╳	✓	✓	✓	✓	✓
Withstands Impersonation Attack	✓	╳	✓	✓	✓	✓
Resists Man-in-the-Middle Attack	╳	╳	✓	✓	✓	✓
Resists Traceability Attack	✓	✓	✓	╳	✓	✓
Offers Perfect Forward & Backward Secrecy	✓	✓	╳	✓	╳	✓

In contrast, the scheme presented by Alghamdi et al. [68] is not safe against MITM, and replay attacks, Algarni et al. [69] is not secure against impersonation and replay attacks, Jan et al. [70] is not offering perfect forward and backward secrecy, Irshad et al. [71] lacks the anonymity and is vulnerable to a traceability attack, and Ghani et al. [72] has a backward secrecy issue. The proposed protocol, on the other hand, is a robust solution, resisting replay, impersonation, MITM, and traceability attacks, and offering perfect forward and backward secrecy.

## Performance analysis

The significant features of the proposed protocol can be measured by considering storage overheads, communication, and computation costs. It is worth mentioning that the proposed protocol has been integrated into the highly reputable MIRACL Crypto SDK [https://miracl.com/]. This cloud-based platform is renowned for its password-less, multi-factor authentication capabilities and is widely used across various works, including [68,69,72,76]. The execution time taken by different cryptographic operations is demonstrated in bullets.

- Hash Cryptographic Function (TH) ≈ 0.149 ms
- ECC Point multiplication (TX) ≈ 0.35 ms
- ECC point addition (T+) ≈ 0.78 ms

The cost of the remaining cryptographic operations is too small, equal to zero, and should be neglected.

### Computation costs.

**Fig 5:**
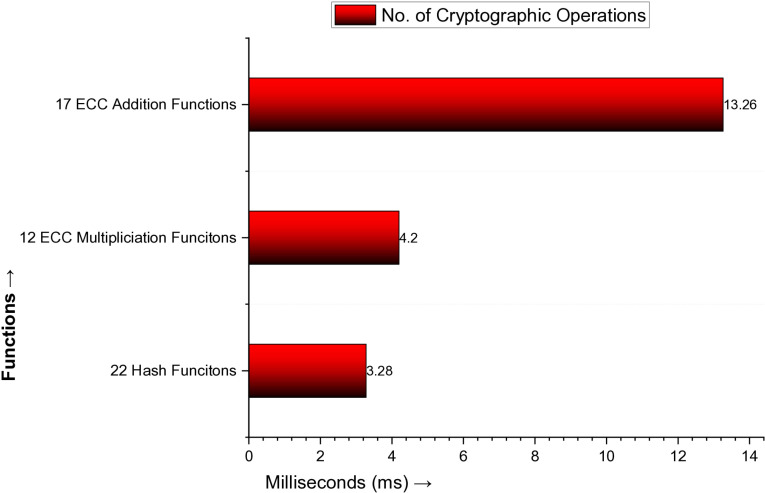
Number of Cryptographic Operations in the Proposed Protocol.

The computation costs are the cryptographic operations in the protocol’s authentication phase. Suppose T_H_ denotes hash cryptographic operations in the authentication phase, point multiplication is T_X_, and point addition is T_+_; xor and concatenation are too small, so we neglect them. Then, the computation costs of the proposed protocol are 22T_H_ + 12 T_X_ + 17T_+_. According to [73], the hash function requires 0.149 ms time to execute, ECC point multiplication is 0.35 ms, ECC point addition is 0.78 ms. By putting these values in the computation cost, i.e., 22T_H_ + 12 T_X_ + 17T_+_ = 22(0.149) + 12 (0.35) + 17(0.78) = 3.28 + 4.2 + 13.26 = 20.74 ms, as plotted in Fig 5.

### Storage overheads.

**Fig 6:**
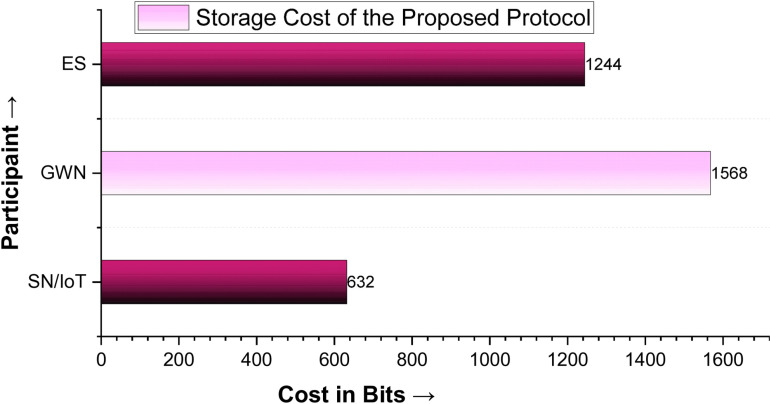
Storage Cost of the Proposed Protocol.

The credentials stored during the registration phase of the protocol are the storage costs. In this regard, SN/IoT device stores {ID_SN_, B_SN_, PW_SN_} = 64 + 512 + 56 = 632 bits, GWN stores {x, y, S_GWN_}, {PK_α_, α, E_P_}, {ID_ES_}, {K_GWN_}, and {C_SN_, D_SN_, I_1_, I_2_, h(.), H(.)} = 32 + 32 + 64 + 160 + 32 + 160 + 64 + 160 + 256 + 256 + 256 + 256 = 1568 bits, and the ES stores {C_SN_, D_SN_, J_SN_, I_1_, I_2_, PK_α_, α} = 256 + 256 + 256 + 256 + 160 + 60 = 1244 bits. By summing up the storage cost of SN, GWN and ES, i.e., 632 + 1568 + 1244 = 3444 bits; therefore, the storage costs of the proposed protocol is 3444 bits as plotted diagrammatically in Fig 6.

### Communication costs.

**Fig 7:**
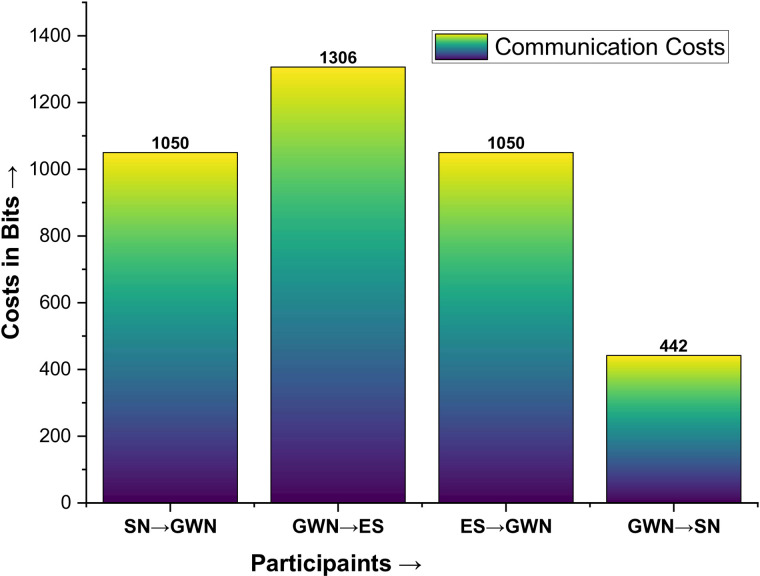
Communication costs.

Exchanging messages among the participants in the authentication phase means communication costs. In this regard, the message communicated between SN and GWN is {E_1_, E_2_, E_3_, E_4_, T}, GWN and ES is {E_5_, E_6_, E_7_, E_8_, E_9_, T}, ES and GWN is {E_10_, E_11_, L_2_, C_SN_, T} and GWN towards SN is {E_10_, β, T}. Again, according to [73], the hash image is 256 bits, the random number is 32 bits, the key is 160 bits, and the timestamp is 26 bits. The communications costs are counted as shown in Table 3 and plotted in Fig 7.

**Table 3:** Communication costs in bits.

Participants	Message	Values	Costs
SN → GWN	{E_1_, E_2_, E_3_, E_4_, T}	256 x 4 + 26	1050
GWN → ES	{E_5_, E_6_, E_7_, E_8_, E_9_, T}	256 x 5 + 26	1306
ES → GWN	{E_10_, E_11_, L_2_, C_SN_, T}	256 x 4 + 26	1050
GWN → SN	{E_10_, β, T}	256 + 160 + 26	442
Total Communication Costs in Bits			3548

### Comparative analysis performance metrics.

**Fig 8:**
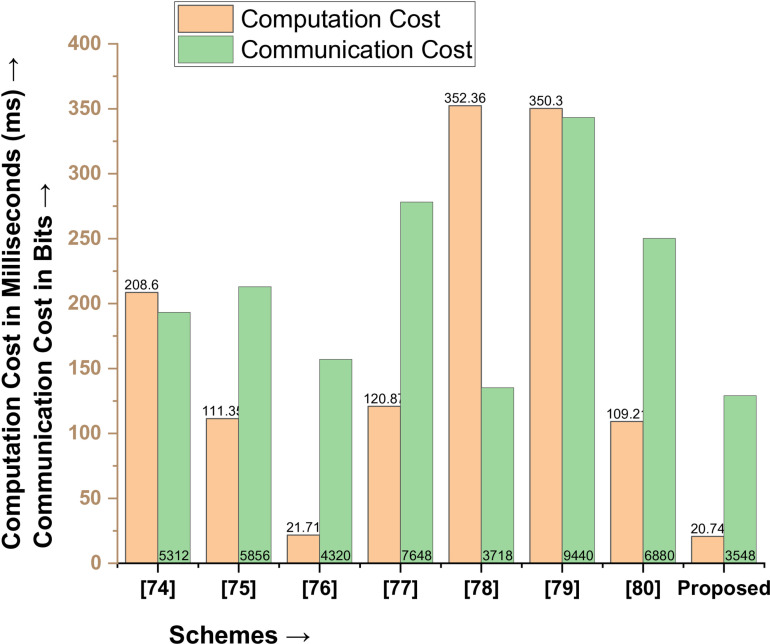
Comparative Analysis in terms of performance metrics.

When comparing the proposed protocol with Mohit et al. [74], Zhou et al. [75], Alzahrani et al. [76], Deebak et al. [77], Krishnasrija et al. [78], Chandrakar et al. [79], and Jia et al. [80], the proposed scheme is outperformed better, as shown in Table 4. The communication cost of Mohit et al. [74] is 5312 bits, and the computation cost is 208.6 ms. This means the proposed protocol is 33.20% better in communication cost and 90.05% in computation cost. The communication cost of Zhou et al. [75] is 5856 bits, and the computation cost is 111.35 ms. This means the proposed protocol is 39.41% better in communication cost and 81.37% in computation cost. The scheme proposed by Alzahrani et al. [76] has a cost of 4320 bits, and computation is 21.71 ms, which means the proposed protocol is 17.87% better in communication and 4.46% in computation cost. Deebak et al. [77] have a communication cost of 7648 bits and a computation cost of 120.87 bits, which means the proposed protocol, is 53.60% better in communication and 82.74% better in computation cost. Overall, the proposed protocol demonstrates its superiority by outperforming all its competitors, as shown in Table 4, and depicted in Fig 8.

**Table 4:** Comparative Analysis (Performance Metrics).

Performance Metrics → Schemes↓	Computation Cost in ms	Communication Cost in Bits
Mohit et al. [74]	208.6	5312
Zhou et al. [75]	111.35	5856
Alzahrani et al. [76]	21.71	4320
Deebak et al. [77]	120.87	7648
Krishnasrija et al. [78]	352.36	3718
Chandrakar et al. [79]	350.3	9440
Jia et al. [80]	109.21	6880
Proposed	20.74	3548

## Conclusion

Securing the edge IoT ecosystem is a critical task, given the unique challenges posed by decentralized data processing and the vulnerabilities of edge servers. A comprehensive approach is needed to address these security concerns effectively. Therefore, this article presents an authentication protocol that utilizes elliptic curve cryptography (ECC), SHA2, and XOR operations. The scheme offers dynamic key computation that strongly resists various attacks, making it suitable for resource-constrained IoT devices. However, it’s crucial to balance security and performance to ensure sustainable operations, which the proposed protocol has, due to random checks in different protocol round trips while maintaining data security and privacy. This balance underscores the urgent need for adaptive security measures in edge environments. The protocol’s robustness has been confirmed through the ROR model, ProVerif simulation, and pragmatic discussion, while the performance metrics have been measured by considering storage, computation, and communication costs. The results from the different analysis sections demonstrated that the proposed protocol is lightweight, robust, and easily implemented for the edge-IoT ecosystem. The same protocol will be used in the future, but the key should be exchanged through modern cryptographic techniques involving AI, ML, and quantum computing; also plan to simulate the security through the Scyther toolkit to fortify the Edge-IoT ecosystem against evolving threats.
